# Culinary memories, perceived barriers, and motivations for home cooking among younger and older adults in Spain: a qualitative cross-sectional study

**DOI:** 10.3389/fnut.2026.1938313

**Published:** 2026-09-24

**Authors:** Alma María Palau-Ferre, Francisca Esteve-Claramunt, Giovanni Cangelosi, Stefano Mancin, Jorge Casaña Mohedo, Valentina Micheluzzi, Elena Sandri, Fabio Petrelli

**Affiliations:** 1 Department of Nutrition and Dietetics, Catholic University of Valencia San Vicente Mártir, Valencia, Spain; 2 Faculty of Medicine and Health Sciences, Catholic University of Valencia San Vicente Mártir, Valencia, Spain; 3 SONEV Research Group, Faculty of Medicine and Health Sciences, Catholic University of Valencia San Vicente Mártir, Valencia, Spain; 4 Department of Nursing, Faculty of Nursing and Chiropody, Universitat de València, Valencia, Spain; 5 School of Pharmacy, Experimental Medicine and “Stefania Scuri” Public Health Department, Camerino, Italy; 6 IRCCS Humanitas Research Hospital, Milan, Italy; 7 Department of Nursing, Catholic University of Valencia San Vicente Mártir, Valencia, Spain; 8 Clinical and Interventional Cardiology, Sassari University Hospital, Sassari, Italy; 9 G-ALIS Research Group, Faculty of Medicine and Health Sciences, Catholic University of Valencia San Vicente Mártir, Valencia, Spain

**Keywords:** behavioral factors, dietary patterns, home cooking, qualitative cross-sectional study, Spain, younger and older adults

## Abstract

**Background/aim:**

Home cooking is a key determinant of health and cultural transmission. In Mediterranean countries like Spain, contemporary social changes are among the factors that may have contributed to shifts in traditional dietary patterns, with young adults potentially more exposed to a dietary transition. This study comparatively explores culinary perceptions, childhood memories, perceived barriers, and daily motivations related to home cooking among young and older adults in Spain.

**Methodology:**

A qualitative, cross-sectional design based on content analysis was conducted across Spain using an online survey administered between November 2025 and January 2026 and reported in accordance with the COnsolidated criteria for REporting Qualitative research tool. To capture intergenerational shifts, two age-based subsamples were extracted from a single overall sample and compared using an extreme case comparison strategy: Young Adults (< 30 years old) and Older Adults (> 59 years old).

**Results:**

From the overall sample of 169 respondents, 78 participants belonging to the two extreme age cohorts were analyzed. Among these, the younger cohort (*n* = 26) experiences culinary habits through the lens of recent independent living, showing a proactive search for less processed foods and a technical modernization of cooking via time-saving appliances (e.g., air fryers) to overcome time constraints. Conversely, the older cohort’s (*n* = 52) practices are rooted in traditional slow cooking. While retirement provides them with abundant time, physical fatigue and loneliness act as barriers. Both generations converge in their emotional attachment to traditional Spanish spoon dishes (“platos de cuchara”) and view socialization as the primary catalyst for cooking.

**Conclusion:**

Participants’ accounts suggest that traditional cooking practices are perceived as a cultural and potential public health resource. Exploring the intergenerational transmission of these practices may contribute to the knowledge base on how healthier and more sustainable food-related behaviors could be fostered, though these findings should be interpreted as hypothesis-generating rather than as direct evidence for policy.

## 1 Background

Food and cooking practices shape dietary patterns throughout the life cycle which, in turn, are key determinants of health, and are themselves closely linked to social, cultural, and behavioral factors. In this sense, home cooking not only influences the nutritional quality of the diet but also represents a central space for socialization, learning, and cultural transmission (1). Evidence has consistently shown that preparing food at home is associated with healthier food choices, higher consumption of fresh produce, and less reliance on ultra-processed foods, reinforcing its role as a key element in promoting public health (2).

In Mediterranean countries like Spain, home cooking has historically been linked to traditional dietary patterns, characterized by the use of fresh ingredients, varied culinary techniques, and a strong sociocultural component. However, in recent decades, social transformations appear to be among the factors associated with changes in these practices. Factors such as urbanization, the reorganization of family roles, entry into the labor market, and changes in lifestyles may have contributed to a gradual dietary transition, marked by a reduction in cooking time and an increase in the consumption of convenience foods that could influence chronic conditions as well (3–5).

This evolution raises important questions about how culinary practices are currently configured and how they vary across generations. The existing literature has shown that young adults are a group particularly vulnerable to these changes (3). During this life stage, characterized by the transition to independence, eating habits are redefined, often influenced by time constraints, a lack of culinary skills, and pressure from academic or work environments (3, 6). However, beyond these structural barriers, recent research suggests that young people’s culinary practices are also influenced by symbolic and relational factors, such as the emotional significance of certain foods, the search for autonomy, or the social dimension of the act of cooking (7).

On the other hand, in the older population, home cooking tends to be more deeply rooted in previous experiences and different sociocultural contexts, where the intergenerational transmission of knowledge played a central role (8). In these groups, culinary practices are often linked to tradition, memory, and cultural continuity, as well as a greater awareness of the role of food in maintaining health (9). In this sense, adherence to traditional dietary patterns, such as the Mediterranean diet, remains an important benchmark, although it is also subject to adaptations depending on changes in health status or living conditions (10).

From a sociocultural perspective, cooking is a complex phenomenon that integrates material, symbolic, and relational dimensions. Culinary memories from childhood, family experiences surrounding food, and the transmission of recipes are key elements in the construction of food identity and in shaping preferences and practices over time (11). However, contemporary social changes may be creating discontinuities in these processes, leading to different relationships with cooking across generations. In this context, it is particularly relevant to explore not only the culinary practices themselves, but also the perceptions, motivations, and barriers that underpin them in different age groups (12). Aspects such as the role of socialization, the emotional value of food, the influence of life trajectory, and the impact of structural conditions (available time, knowledge, resources) can shape the culinary experience differently for young and older adults (13).

Despite the growing interest in the relationship between cooking, health, and culture, comparative qualitative evidence across generations remains limited, especially in the Spanish context (4). In particular, there is a gap in the integrated understanding of how childhood memories, family experiences, perceived barriers, and everyday motivations influence the practice of home cooking at different stages of life (14). Therefore, the present study aims mainly to comparatively explore culinary perceptions, childhood memories, perceived barriers, and everyday motivations related to home cooking among young and older adults in Spain.

## 2 Methods

### 2.1 Study design and reporting

The present research employed a qualitative, cross-sectional design based on the qualitative content analysis of open-ended survey responses, following the principles established by Graneheim and Lundman (15), which constituted the dominant analytical framework guiding the entire coding process. The use of an online survey as a legitimate tool for qualitative data collection was supported by the methodological approach described by Braun et al. (14). This combination was chosen for its ability to explore participants’ perspectives in depth while achieving broad population coverage and ensuring consistency across responses. The manuscript was reported according to the Consolidated Criteria for Reporting Qualitative Research (COREQ) (16) guidelines (Checklist in Supplementary material S1). Throughout this manuscript, “home cooking” refers to the practice of preparing meals at home, whereas “traditional cooking” denotes the specific repertoire of culturally transmitted Mediterranean dishes and techniques. The two terms are conceptually distinct and were defined *a priori*; where the findings link them, this reflects participants’ own association of home cooking with traditional culinary heritage.

### 2.2 Ethical considerations

The research protocol was fully reviewed and approved by the Research Ethics Committee of the Universidad Católica de Valencia San Vicente Mártir (Approval code: UCV/2025–2026/011, issued on November 3, 2025). All procedures were conducted in strict accordance with the ethical standards outlined in the Declaration of Helsinki (17). Prior to accessing the questionnaire, all respondents were provided with detailed information regarding the study’s purpose, and the voluntary nature of their involvement. Electronic informed consent was mandatory to proceed, and all collected data were anonymized to guarantee privacy and confidentiality according to the European Legislation for personal data protection (18).

### 2.3 Procedure, data collection and enrollment

Consistent with the analytical framework described above, data were collected using an online survey administered between November 2025 and January 2026. To capture the narratives surrounding home cooking and its role in disease prevention within family life, data were collected using an online survey administered between November 2025 and January 2026. The questionnaire was developed following an extensive literature review of previous studies (7, 19–23) and subsequent adaptation to the local context. To guarantee its suitability for qualitative content analysis, the tool was thoroughly reviewed and refined by two experts in the field of qualitative and topic-specific research (a university professor and a Public Health PhD researcher). The survey produced combined socio-demographic elements that preliminary stratified the sample for possible inclusion in the observation for age. Profiling with a core section of five open-ended questions: four key questions, and one final question (Supplementary material S2). These prompts were specifically designed to elicit detailed accounts regarding the importance of sharing meals, the perception of cooking as an act of caregiving, the role of home cooking in preventing diet-related diseases, recent modifications in culinary habits, opinions on school-based cooking education, and attitudes toward ready-made or takeaway food. For transparency, the five open-ended questions were, in summary: (1) In your childhood, what did cooking and eating at home mean to you, and how have your culinary habits evolved since then? (2) Which dish or dishes are unforgettable or indispensable for you, and why? (3) What are the main barriers and facilitators you currently experience when cooking at home? (4) What motivates you to cook, and what would help you cook more often? (5) What is your opinion on ready-made or takeaway food and on introducing cooking education in schools?

#### 2.3.1 Dissemination

To achieve a broad, heterogeneous, and cost-effective sample across Spain, the research team implemented a multi-channel digital dissemination strategy, an approach increasingly validated for qualitative and health-related research seeking wide geographical reach at low cost (14). The survey link was distributed through various social and professional networks (including Facebook, Instagram, and LinkedIn). This digital approach was reinforced by physical, on-the-ground recruitment efforts supported by university students enrolled in the Human Nutrition and Dietetics degree program. Participants completed the survey autonomously, and the system restricted submissions to one per user to prevent duplicated data. Because the survey was disseminated openly, eligibility was verified after data collection rather than through prior screening. Once the data collection period concluded, the responses were exported and screened against the inclusion criteria: being an adult resident in Spain, having provided informed consent, and having completed the open-ended section with responses relevant to the core topics. Applying eligibility criteria a posteriori entailed a modest additional processing step but did not compromise data collection, as the single-submission restriction and the structured survey design minimized invalid entries. A formal comparison between eligible and non-eligible respondents was not feasible, since excluded responses generally lacked sufficient data for characterization. The remaining valid responses were then prepared for the comparative coding of the two target generational cohorts (participants under 30 and over 59 years old).

### 2.4 Sample

The age-based stratification applied in this study constituted an *a priori* analytical decision rather than a *post hoc* grouping: the two extreme age cohorts were defined before the analysis, as part of the extreme case comparison strategy, and the specific thresholds were established on theoretical grounds (detailed in Section 2.4.1). Accordingly, while the source survey captured responses from adult participants of all ages, only two purposively defined subsamples were extracted from this single, Spain-wide dataset to evaluate intergenerational shifts in culinary practices: a younger cohort of adults under 30 years of age, and an older cohort of adults over 59 years of age.

Consistent with the screening described in Section 2.3.1, participants whose responses were incomplete, excessively brief, or lacked relevance to the core topics were excluded from the final qualitative analysis. A response was operationally considered to lack relevance to the core topics when it did not address any of the five open-ended questions, for instance, off-topic remarks, single-word answers with no reference to cooking practices, memories, barriers, or motivations, or content unrelated to home cooking. This relevance criterion was applied consistently throughout the analysis, including the comparative stage reported in Section 3.7.

The initial source sample comprised 169 respondents with a mean age of 49.6 years (SD = 15.0; range 18–77), of whom 74.0% were women. Most respondents lived in Madrid (33.1%), followed by Valencia, Valladolid, and Burgos. Educational attainment was generally high, with university graduates representing the largest group (39.6%). Approximately one-third of participants (32.5%) reported a background in health-related disciplines. The detailed characteristics of this source sample are presented in Table 1.

**Table 1:** Participant characteristics.

Characteristic	*n* (%) or statistic
Total participants	169
Age, years, mean (SD)	49.6 (15.0)
Age, years, median (range)	54 (18–77)
Age group, *n* (%)	
18–29	26 (15.4)
30–39	14 (8.3)
40–49	28 (16.6)
50–59	49 (29.0)
60–69	47 (27.8)
≥ 70	5 (3.0)
Gender, *n* (%)	
Female	125 (74.0)
Male	44 (26.0)
Place of residence, *n* (%)	
Madrid	56 (33.1)
Valencia	33 (19.5)
Valladolid	24 (14.2)
Burgos	23 (13.6)
Other provinces	33 (19.5)
Educational level completed, *n* (%)	
Primary education	1 (0.6)
Lower secondary education	3 (1.8)
Upper secondary education	23 (13.6)
Intermediate vocational training	6 (3.6)
Higher vocational training	27 (16.0)
University degree	67 (39.6)
Master’s degree	26 (15.4)
Doctorate	16 (9.5)
Health-related studies, *n* (%)	
No health-related studies	114 (67.5)
Health-related studies	55 (32.5)

#### 2.4.1 Subsampling strategy and generational rationale

The threshold of < 30 years was selected based on established European demographic and sociological indicators regarding delayed emancipation and late-stage family formation (24). In Spain, this cohort encompasses “Generation Z,” a demographic group heavily characterized by high rates of residential dependence, late entry into independent household management, and documented barriers regarding cooking self-efficacy and time-scarcity. Furthermore, their dietary schema is highly influenced by digitalized public health discourses, abstract nutritional literacy, and technology-driven food environments (4).

Conversely, the > 59 years threshold aligns with the sociological and epidemiological transition toward retirement and senior life stages in Southern Europe. Born between the 1950s and mid-1960s, these participants represent the historical baseline of domestic culinary transmission in Spain, having been socialized in environments dominated by raw, fresh ingredients, shorter food supply chains, and traditional gender-segregated domestic labor (1, 25). Epidemiologically, this threshold represents an intersection where food choices shift from being proactively wellness-oriented to reactively medicalized, often driven by the clinical necessity to manage age-related chronic pathologies (e.g., hypertension, hypercholesterolemia) through traditional Mediterranean dietary patterns.

### 2.5 Data analysis

The textual corpus generated by the selected cohorts was subjected to a mixed inductive-deductive qualitative content analysis (15). The four main categories (childhood memories and culinary evolution; memorable and indispensable dishes; barriers and facilitators; and motivations) were deductively informed by the thematic structure of the open-ended questions, which had themselves been derived from the literature; this constituted a deliberate directed content-analysis choice rather than an artifact of the process. Within this predefined categorical frame, the subcategories were developed inductively from the data. Following a thorough process of familiarization with the raw data, researchers identified relevant text segments and isolated them as meaning units. These units were subsequently condensed and assigned preliminary codes. Through an iterative process of comparison and refinement, these codes were grouped into broader subcategories and overarching main categories that highlighted the generational convergences and divergences regarding home cooking. This directed approach ensured coherence between the research objectives and the analytical output while preserving inductive openness at the subcategory level.

### 2.6 Trustworthiness

To ensure the methodological soundness of this qualitative comparative analysis, the study strictly adhered to the four criteria of trustworthiness established by Lincoln and Guba (25). First, credibility was safeguarded by implementing a highly systematic coding procedure, which involved repeated, immersive readings of the textual corpus. Researcher triangulation was utilized to debate findings and resolve discrepancies, and direct, literal participant quotations were deeply embedded into the results to firmly anchor the interpretations in the raw data.

Second, transferability was facilitated by providing an exhaustive account of the research context, the specific demographic characteristics of the two selected age cohorts, and the step-by-step analytical process, thereby allowing other researchers to evaluate the applicability of these findings to different contexts or populations.

Third, dependability was ensured by meticulously documenting every stage of the research process, creating a comprehensive analytical audit trail that tracked the transformation of raw meaning units into definitive intergenerational themes.

Finally, confirmability was promoted by maintaining a clear, transparent link between the participants’ original responses and the final categories, alongside continuous team discussions designed to reduce individual biases during the interpretation phase.

### 2.7 Thematic sufficiency

Because the data were collected through a cross-sectional online survey, the recruitment process was not guided by traditional, iterative data saturation, a criterion whose applicability to this type of qualitative data has been increasingly debated (26). Instead, the adequacy and richness of the data were evaluated retrospectively by assessing thematic sufficiency, an approach consistent with the concept of information power, whereby samples holding rich, relevant information require fewer participants to yield analytically sufficient data (27). Operationally, the research team chronologically ordered the complete corpus of responses by submission date and examined them sequentially in that order. The earliest responses from both age cohorts laid the foundation for the primary analytical dimensions. As the review progressed through the chronologically ordered corpus, the point at which subsequent responses ceased to add new analytical dimensions, adding only depth, variation, and nuance to the already established categories, was documented as the indicator of thematic sufficiency. This confirmed that the gathered data provided sufficient informational power and conceptual thickness to comprehensively address the research objectives regarding generational shifts in cooking practices.

### 2.8 Analytical stability

Building upon the assessment of thematic sufficiency, analytical stability was verified to further assess the stability and robustness of the coding framework. As the final blocks of responses from the targeted participants were analyzed, researchers observed that these later inputs consistently corroborated the already established categories and could be accommodated within the existing coding framework without requiring modification of its subcategories or core thematic structure. Rather than invoking data saturation, a concept whose coherence and usefulness have been questioned in contemporary qualitative methodology (26), this indicated that the coding framework had reached stability, in the sense that its analytical structure remained coherent and sufficient to organize the incoming data.

Nevertheless, all valid responses from the selected cohorts were retained and fully integrated into the final analysis to enhance descriptive richness and provide a wider, more diverse array of illustrative quotations for the intergenerational comparison. To further guarantee analytical stability and protect against individual researcher bias during the coding of textual data, a parallel blind-coding process was conducted on a subset of the responses by the two qualitative research experts mentioned in the data collection procedure. Inter-rater discrepancies regarding preliminary subcategories were systematically discussed and resolved through iterative consensus meetings until a final, unified thematic structure was achieved.

## 3 Results

### 3.1 Participant characteristics

From the initial pool of survey respondents, 78 participants met the criteria for the two extreme age cohorts and constituted the analytical sample of this study. The characteristics of the total source sample are reported in the Methods section and are not reiterated here; the present section focuses on the analyzed cohorts.

### 3.2 Stratified characteristics of sample

The age-stratified analysis included 26 participants in the younger cohort (< 30 years) and 52 in the older cohort (> 59 years), representing 15.4 and 30.8% of the total sample, respectively. As expected, the mean age was 22.1 years [Standard Deviation (SD) 2.9] in the younger group and 64.6 years (SD 4.1) in the older group. Women predominated in both cohorts, accounting for 76.9% of the younger participants and 61.5% of the older participants. Madrid was the most common place of residence in both age groups (53.8 and 44.2%, respectively).

Compared with the younger cohort, a higher proportion of older participants resided in Valladolid (23.1% vs. 3.8%). Educational attainment differed between age groups. In the younger cohort, upper secondary education and university degrees were the most frequent qualifications (both 30.8%), followed by higher vocational training (23.1%). In contrast, university degrees were the most common qualification among older participants (44.2%), and a notable proportion held a master’s degree or doctorate (13.5% each), whereas no doctoral degrees were reported in the younger group.

Participants in the younger cohort were more likely to have a health-related educational background than those in the older cohort (57.7% vs. 30.8%). Conversely, the majority of older participants reported no health-related studies (69.2% vs. 42.3% in the younger cohort). These findings suggest differences between generations in educational profiles and exposure to health-related training. See summary in Table 2.

**Table 2:** Socio-demographic characteristics of the cohorts included.

Characteristic	Younger Cohort (< 30 years)	Older Cohort (> 59 years)
Total participants, *n* (%)	26 (15.4)	52 (30.8)
Age, years, mean (SD)	22.1 (2.9)	64.6 (4.1)
Age, years, median (range)	22 (18–29)	64 (60–77)
Gender, *n* (%)		
Female	20 (76.9)	32 (61.5)
Male	6 (23.1)	20 (38.5)
Place of residence, *n* (%)		
Madrid	14 (53.8)	23 (44.2)
Valencia	3 (11.5)	6 (11.5)
Valladolid	1 (3.8)	12 (23.1)
Burgos	0 (0.0)	1 (1.9)
Other provinces	8 (30.8)	10 (19.2)
Educational level completed, *n* (%)		
Primary education	1 (3.8)	0 (0.0)
Lower secondary education	0 (0.0)	2 (3.8)
Upper secondary education	8 (30.8)	6 (11.5)
Intermediate vocational training	0 (0.0)	1 (1.9)
Higher vocational training	6 (23.1)	6 (11.5)
University degree	8 (30.8)	23 (44.2)
Master’s degree	3 (11.5)	7 (13.5)
Doctorate	0 (0.0)	7 (13.5)
Health-related studies, *n* (%)		
No health-related studies	11 (42.3)	36 (69.2)
Health-related studies	15 (57.7)	16 (30.8)

SD, standard deviation.

### 3.3 Descriptive analysis of younger participants (< 30 years old)

The qualitative analysis of participants under 30 years of age revealed four main categories, each divided into distinct subcategories that deeply illustrate their current culinary habits and perceptions.

#### 3.3.1 Childhood memories and culinary evolution

As these young adults transition to independent living (often in university residences or their first apartments) most became increasingly conscious of their diets. Many reported a deliberate shift away from the pre-made or ultra-processed foods (UPF) of their childhood, striving for fresher, healthier, and more balanced meals as a hallmark of their newfound autonomy and self-care. Alongside this, while several fondly remembered the traditional meals prepared by their parents, their current culinary practices were highly modernized: a noticeable reduction in heavy frying was accompanied, for a majority, by a preference for efficient, health-conscious techniques such as grilling, baking, and the widespread use of new appliances like air fryers and microwave steamers.

#### 3.3.2 Memorable and indispensable dishes

When asked about unforgettable flavors, many in this demographic cited specific family recipes, such as a grandmother’s pasta, a father’s omelet, or a mother’s chili—meals deeply intertwined with emotional memories representing care, nostalgia, and family unity. Despite their fast-paced lifestyles, most also showed a surprisingly strong attachment to traditional Spanish spoon dishes (platos de cuchara) like lentil stews, paella, and cocido, which they viewed as the ultimate comfort food that grounds them, brings a sense of “home,” and provides affordable nutrition.

#### 3.3.3 Current barriers and facilitators

The most prominent barrier to cooking for this group was the sheer scarcity of time. For most participants, hectic schedules filled with university classes, part-time jobs, and early-career demands relegated cooking to a secondary priority, leading to frustration due to poor time management and limited culinary knowledge. To counter this, many relied heavily on modern kitchen technology: appliances like air fryers, microwaves, and silicone steaming cases, together with prior organization and semi-prepared ingredients, acted as crucial facilitators, allowing them to prepare relatively healthy meals quickly and efficiently.

#### 3.3.4 Motivations and aids for cooking

Cooking was rarely enjoyed as a solitary activity for this group. For most, the primary motivator was socialization: preparing a meal for a partner, cooking with friends, or sharing a lunch transformed the act of cooking from a daily chore into a meaningful social event and a practical expression of love. When asked what would help them cook more often, the overwhelming response among participants was having more free time; several additionally viewed achieving full residential and economic independence as a key milestone that would naturally foster better, more frequent cooking habits.

### 3.4 Qualitative results: quotes by category and subcategory (< 30 years old)

The qualitative analysis of participants under 30 years of age (Table 3) reveals significant shifts in culinary habits, structured around four main thematic categories.

**Table 3:** Detailed qualitative results < 30 years old group.

Category	Subcategory	Literal response from participant
1. Childhood memories and culinary evolution	3.3.1.1 Search for independence and less processed food	“When I was little, they used to cook me many pre-made products… Now I live alone and cook for myself, so I try to make everything as least ultra-processed as possible.”
		“First and foremost, it was fresh food, made in the moment… Now, due to my status as a student in a Residence Hall… it is varied, balanced, and healthy.”
		“I remember eating everything when I was little and I loved it, but over time I started eating worse… Now that I live alone I do not eat as well.”
	3.3.1.2 Modernization of techniques and use of appliances vs. tradition	“While previously frying was used more and vegetables were barely included, currently techniques like grilling are prioritized.”
		“My childhood home and my current one are the same… Currently, we have also added cooking with an air fryer, a method we now use regularly.”
		“Food was baked, grilled, boiled, and fried. Currently, there is less fried food.”
2. Memorable and indispensable dishes	3.3.2.1 Family tradition and dishes with affective meaning	“Macaroni bolognese, a staple throughout my life, easy and quick to prepare.”
		“Mexican food has always been one of our favorites, the chili con carne my mother makes is one of her best recipes.”
		“My father’s Spanish potato omelet, a perfect omelet, without being excessively set.”
	3.3.2.2 Stews and comforting spoon dishes	“Lentils and ‘cocido’ (stew). I do not know why, but I find them homey.”
		“Legume stews. Because I love legumes, they are healthy and affordable.”
		“The Sunday paella at my parents’ house.”
3. Current barriers and facilitators	3.3.3.1 Lack of time and stress from studies/work	“My culinary ignorance and the little time I have between studies and work make it difficult for me.”
		“Many times I have to leave quickly to go to university and have a busy morning, so I often change the recipe to a quicker one due to lack of time.”
		“Time and also motivation… Many times I have to leave quickly.”
	3.3.3.2 Modern tools and quick solutions as facilitators	“Now I have an air fryer that helps me a lot. I do not usually have much time to cook so I make quick and easy things.”
		“The microwave and silicone cases for steaming… help me a lot to cook healthy, tasty, and fast.”
		“Prior organization makes it easier. Lack of time and knowledge make it difficult.”
4. Motivations and aids for cooking	3.3.4.1 Socialization with partner, friends, and family	“It is very important to eat accompanied, because sometimes that is the only moment you see those people.”
		“I like to cook for my partner often, since sharing that moment is special to me.”
		“Yes, if I am going to eat or even cook with someone, I am always more motivated.”
	3.3.4.2 The need for more time availability and independence	“Having more free time.”
		“Becoming independent.”
		“Having time and schedule availability.”

Regarding the first category - Childhood Memories and Culinary Evolution - the transition to independent living marks a turning point where young adults actively seek a diet with fewer processed foods. For instance, one participant highlighted this shift by stating that while she used to eat many pre-made products as a child, she now lives alone and tries “to make everything as least ultra-processed as possible”. Another university student residing in a college hall reflected on the evolution from the fresh, homemade food of his childhood to a diet that remains “varied, balanced, and healthy”. However, this transition can be challenging, as some acknowledge that while they used to eat well during their childhood, living independently has caused them to “not eat as well”. Taken together, these accounts show that the category Childhood Memories and Culinary Evolution is not merely descriptive but marks independent living as the pivotal moment that reactivates a conscious, health-oriented renegotiation of inherited eating patterns, thereby anchoring the theme of generational culinary transition.

Accompanying this search for healthier options is a clear modernization of culinary techniques. Younger participants report a significant reduction in traditional heavy frying, noting that “while previously frying was used more and vegetables were barely included, currently techniques like grilling are prioritized.” The introduction of new appliances has notably altered daily routines, with participants mentioning the addition of “cooking with an air fryer, a method we now use regularly.” These accounts reinforce that culinary evolution in this cohort operates through technological substitution rather than rupture: traditional dishes are retained while their preparation is modernized, illustrating how the category captures continuity and change simultaneously.

When exploring the second category - Memorable and Indispensable Dishes - the affective value of family traditions becomes deeply evident. Many young adults remain strongly attached to dishes that symbolize care, nostalgia, and family unity. One participant fondly recalled her mother’s international cooking, noting that “*the chili con carne my mother makes is one of her best recipes*.” Others associate comfort with simple, staple meals, describing dishes like “*macaroni bolognese, a staple throughout my life, easy and quick to prepare*,” or “*my father’s Spanish potato omelet, a perfect omelet, without being excessively set*.”

Furthermore, traditional Spanish spoon dishes continue to hold a special place in their culinary memory. Despite modern fast-paced lifestyles, stews and legumes are viewed as the ultimate comfort food. Participants express a strong preference for “lentils and ‘cocido’ (stew),” simply because they “find them homey.” These legume stews are highly valued not only for their taste but also because “they are healthy and affordable,” while others nostalgically look back at family gatherings centered around “the Sunday paella at my parents’ house.” Collectively, these quotations demonstrate that Memorable and Indispensable Dishes functions as an affective anchor, whereby spoon dishes consolidate the link between emotional memory, affordability, and cultural identity that defines the theme.

In terms of the category - Current Barriers and Facilitators - the main obstacles to cooking for this demographic are a lack of culinary knowledge and severe time constraints imposed by academic or professional lives. One young woman explicitly stated that her “*culinary ignorance and the little time I have between studies and work make it difficult for me*.” Hectic mornings often dictate their culinary choices, with another student admitting that she frequently has “*to leave quickly to go to university and have a busy morning, so I often change the recipe to a quicker one due to lack of time*.”

To counter these barriers, modern kitchen tools act as essential facilitators. The widespread adoption of the air fryer is a prime example; as one participant noted, having an air fryer helps a lot because she does not “usually have much time to cook so I make quick and easy things.” Other time-saving devices like “the microwave and silicone cases for steaming” are highly praised for helping young adults “cook healthy, tasty, and fast.” Ultimately, success in the kitchen hinges on “prior organization,” which facilitates the process, whereas the “lack of time and knowledge make it difficult.” These statements evidence that, for younger adults, barriers and facilitators are two sides of the same structural constraint: modern appliances are valued precisely because they neutralize the time scarcity that otherwise governs the category.

Finally, regarding -Motivations and Aids for Cooking - socialization emerges as the primary driver among the younger generation, transforming meal preparation from a solitary chore into a meaningful social event. One participant emphasized that “it is very important to eat accompanied, because sometimes that is the only moment you see those people.” This sentiment is echoed by those who view cooking as an expression of affection, stating, “I like to cook for my partner often, since sharing that moment is special to me.” Simply having company acts as a catalyst, as another young woman confessed that she is “always more motivated” if she is going to eat or cook with someone. When asked what would help them cook more frequently, the overwhelming consensus points to “having more free time.” Achieving residential milestones such as “becoming independent” and securing more “time and schedule availability” are seen as the ultimate aids that would enable them to establish better, more consistent culinary habits. Overall, these accounts confirm that within Motivations and Aids for Cooking, socialization operates as the decisive catalyst that reframes cooking as a relational act, while structural autonomy is perceived as the condition enabling its consolidation.

### 3.5 Descriptive analysis of older participants (> 59 years old)

The qualitative analysis of participants over 59 years of age revealed four main categories, each divided into distinct subcategories that illustrate a culinary reality shaped by tradition, the aging process, and the value of socialization.

#### 3.5.1 Childhood memories and culinary evolution

The childhood memories of this older demographic were deeply rooted in traditional, slow-cooked meals made with basic, locally sourced ingredients. For the oldest participants, these memories also reflected an era of post-war scarcity, contrasting sharply with the abundance of modern supermarkets. While most preserved the essence of their family recipes, their current cooking habits had evolved significantly: many now prioritized health by reducing salt, heavy fats, and caloric density, seamlessly integrating modern kitchen appliances to achieve the traditional flavors of their youth with much less physical effort.

#### 3.5.2 Memorable and indispensable dishes

The most indelible culinary memories for most of this group belonged to platos de cuchara (spoon dishes) such as lentil stews, Madrid-style cocido, and legume-based meals. These dishes were considered functionally indispensable for their nutritional value but, more importantly, served as the ultimate symbol of maternal care and domestic warmth. For many, the concept of “fiesta” and family unity was inextricably linked to specific festive recipes: memories of Christmas Eve dinners, roasted meats, or elaborate seafood dishes remained highly vivid, representing the joy of a household coming together during their childhood.

#### 3.5.3 Current barriers and facilitators

Unlike the younger cohort, most of these participants identified the abundant free time derived from retirement as their greatest culinary facilitator. However, for several this advantage was counterbalanced by physical fatigue, mobility issues, or the natural barriers associated with aging. To compensate for a lack of energy or to simplify the process, many had widely embraced modern technology: pressure cookers, induction hobs, and food processors (like Thermomix) or air fryers were frequently cited as essential tools that allowed them to maintain a high standard of home cooking without the exhausting labor of the past.

#### 3.5.4 Motivations and aids for cooking

Cooking for this demographic was overwhelmingly a social endeavor. For most, preparing meals for just one or two people was perceived as monotonous or impractical, so hosting guests, receiving family members, or organizing gatherings were the primary motivators that encouraged them to spend time in the kitchen. Beyond mere nutrition, many viewed cooking as an active demonstration of love and a tool for family cohesion: preparing a beloved recipe for their children or grandchildren was a deeply satisfying act, allowing them to care for their loved ones while keeping family traditions alive.

### 3.6 Detailed qualitative results: narrative analysis of older participants (> 59 years old)

The qualitative analysis of participants over 59 years of age (Table 4) is also structured around four main thematic categories.

**Table 4:** Detailed qualitative results > 59 years old group.

Category	Subcategory	Literal response from participant
1. Childhood memories and culinary evolution	3.5.1.1 Slow cooking, with basic and garden ingredients	“It was a simple, traditional, and balanced cuisine. There is little difference.”
		“I remember my mother’s stews… There was a lot of spoon food, legumes, rice… she went shopping every day and brought fresh products.”
		“It was a basic cuisine with village influence and generally affordable ingredients… at night there were very frequently soups.”
	3.5.1.2 Current adaptation toward lighter and healthier preparations	“I have made changes to care for older people and for myself. I include quite a lot of vegetables and meat and fish, almost always grilled. I do not usually cook fried or fatty foods.”
		“I cook less and less fried food and more boiled, steamed, grilled, or baked.”
		“I reduced salt in all my dishes, I do not even try white sugar… I take very little salt.”
2. Memorable and indispensable dishes	3.5.2.1 Stews, legumes, and maternal heritage	“Croquettes, lentils, meatballs, remind me of the ones made at home and I still like them.”
		“Potatoes ‘a la importancia’, squid in its ink, larded veal round.”
		“Yes, almost all of them: stewed lentils, Madrid-style stew (cocido), ham croquettes, Spanish potato omelet…”
	3.5.2.2 Flavors associated with family celebrations and festivities	“Beef in sauce. It reminds me of Christmas because my mother used to make it ahead of time since she did not want to be in the kitchen that day.”
		“The boiled eggs that my mother made at Christmas, with peppers and mayonnaise… the memory of that flavor generates nostalgia.”
		“Squid in its ink. My grandmother made them deliciously… it was the Christmas Eve party.,”
3. Current barriers and facilitators	3.5.3.1 Free time derived from retirement stage	“I am retired and I have plenty of time.”
		“It is made easier by the fact that I am retired. What makes it difficult is that I do not know how to cook.”
		“Since I am early-retired I have time to cook and facility to get the food… There is only one problem: I do not like cooking.”
	3.5.3.2 Use of technology to speed up or compensate for age	“The ceramic hob, the oven, the microwave, the air fryer, the Thermomix. I try to cook three times a week.”
		“Facilitates: oven, pressure cooker, ceramic hob. Age makes it difficult.”
		“The pressure cooker, the induction cooker for its speed. I have nothing that makes cooking difficult.”
4. Motivations and aids for cooking	3.5.4.1 Socialization and motivation when hosting	“Sharing it with others… I think that is my true motivation because otherwise I would not cook.”
		“Having guests at home. Cooking for a single person means you cook the same dish for more than one time.”
		“When my children come, lunch and dinner are meeting and chatting points and we take the opportunity to make the food they like.”
	3.5.4.2 Direct care for health and personal well-being	“Caring for others and caring for myself. I increasingly find my food, without expectations, tastier than what they offer me outside.”
		“Of course. Food at home is infinitely healthier.”
		“I have never seen it that way, for me it is not an act of caring greater than washing… although it is true that cooking is much more direct to health.”

#### 3.6.1 Childhood memories and culinary evolution

For the older generation often revolve around traditional slow cooking. For some of the most senior participants, these memories reflect an era of culinary survival and extreme scarcity. As one participant vividly described their experience, the evolution to modern times is a matter of “*survival, it cannot be compared, from nothing to everything*.” Others recall an environment where food preparation was highly structured and treated with immense care. Reflecting on this, an older woman noted how “*food was cared for a lot. In general, the first courses were spoon dishes and the second was fish or meat, and at night sometimes eggs. Now less time is dedicated to cooking*.” Despite this deep nostalgia, their current practices demonstrate a strong, conscious adaptation toward healthier lifestyles. Detailing this transition, another interviewee explained: “*I have positive memories of dishes stewed with time and affection by my mother. Now I try to cook her recipes, adapted to our way of life and not as caloric*.” These narratives show that, for the older cohort, *Childhood Memories and Culinary Evolution* is structured around a tension between scarcity-rooted nostalgia and a deliberate health-conscious reformulation, whereby maternal recipes are preserved in essence but consciously adapted, anchoring the theme in continuity through adaptation.

#### 3.6.2 Memorable and indispensable dishes nostalgia

Plays a crucial role in shaping the culinary preferences of this demographic, with festive meals and hearty stews holding the utmost emotional value. Unforgettable dishes are frequently intertwined with special family celebrations. This is exemplified by an older adult who highlighted a specific dish, explaining: “Beef in sauce. It reminds me of Christmas because my mother used to make it ahead of time since she did not want to be in the kitchen that day and wanted to enjoy her children.” Similarly, the sensory memory of festive gatherings remains vivid for another participant who nostalgically recalled “the squid in its ink. My grandmother made them deliciously. The cardoon with pine nut sauce. It was the Christmas Eve party, as well as the almond soup.” Beyond holiday celebrations, classic Spanish spoon dishes and deeply rooted traditional recipes remain an indispensable part of their identity. Many proudly highlight meals like “potatoes ‘a la importancia’, squid in its ink, larded veal round, and sea bream in green sauce” as the fundamental culinary anchors of their lives. Taken together, these quotations evidence that Memorable and Indispensable Dishes operates as a repository of collective family memory, where festive and spoon dishes function less as nutrition than as symbolic markers of identity and belonging that sustain the theme.

#### 3.6.3 Barriers and facilitators

Retirement emerges as the most transformative factor in the culinary routines of older adults. For many, the end of their working life provides a significant advantage, with some explicitly stating, “I am retired and I have plenty of time.” However, having the gift of time does not universally equate to a desire to cook. As one early-retiree confessed: “Since I am early-retired I have time to cook and facility to get the food I need in my neighborhood. There is only one problem: I do not like cooking. Although what I really do not like is having to make and think about lunch and dinner every day.” Conversely, for those who do wish to cook, physical limitations associated with aging—such as the simple fact that “fatigue is bad for cooking”—are frequently cited as primary barriers that make daily kitchen tasks increasingly difficult. To successfully compensate for this decline in energy, the older demographic relies heavily on modern household technology. They frequently praise the immense convenience of appliances, noting how “the ceramic hob, the oven, the microwave, the air fryer, the Thermomix” or “the pressure cooker and the induction cooker for their speed” drastically accelerate their routines and reduce the physical burden of cooking. These accounts demonstrate that within Barriers and Facilitators, retirement time is a necessary but insufficient facilitator: its culinary value is mediated by physical capacity and personal motivation, and technology emerges as the compensatory mechanism that reconciles the two, giving the category its distinctive intergenerational contrast.

#### 3.6.4 Motivations and aids for cooking

Finally, socialization acts as the absolute driving force for cooking among older adults. Preparing elaborate meals for a single person is frequently seen as an impractical and monotonous chore. As one participant pointed out, having guests at home is essential because “cooking for a single person means you cook the same dish for more than one time and you have to find how to preserve them so as not to repeat for several days.” The act of hosting transforms cooking from a daily necessity into a deeply meaningful event. Older adults relish the moments when their families visit, viewing these gatherings as vital anchors: “When my children come, lunch and dinner are meeting and chatting points and we take the opportunity to make the food they like. In those moments I cook more.” Ultimately, for many in this demographic, the greatest motivation to step into the kitchen is the joy and validation they feel when others appreciate their efforts. As one participant beautifully summarized, their true motivation simply comes from having “friends and acquaintances ask me for it,” turning the culinary experience into a profound expression of connection, generosity, and affection. Overall, these statements confirm that Motivations and Aids for Cooking is governed by a relational logic: for older adults, cooking derives its meaning from being shared and appreciated, so that socialization simultaneously prevents isolation and validates culinary heritage, consolidating the theme.

### 3.7 Comparative analysis: intergenerational culinary perspectives (< 30 vs. > 59 years old)

A comparative analysis between the younger and older cohorts reveals distinct generational pathways characterized by specific points of convergence and divergence.

#### 3.7.1 Key similarities (convergences)

Both generations share a profound emotional connection to traditional Spanish spoon dishes (*platos de cuchara*, such as lentils and *cocido*), viewing them as comfort food associated with family identity. Furthermore, socialization is the primary catalyst for cooking in both cohorts. For younger adults, preparing food with a partner or friends transforms a daily chore into a social event. For older adults, cooking acts as a vital mechanism to avoid isolation, serving as an opportunity to host family and preserve culinary heritage. Notably, both cohorts rely on similar modern kitchen appliances (e.g., air fryers and microwave ovens) as key facilitators, albeit for different reasons.

#### 3.7.2 Key differences (divergences)

The primary divergence lies in their transition of habits and the nature of their cooking barriers. For young adults, current practices are defined by a search for autonomy and a proactive attempt to decrease ultra-processed food intake following recent independent living. Their main barriers are structural, specifically, lack of time due to academic/work routines and limited culinary knowledge. In contrast, the older generation represents a transition from traditional slow-cooked methods (often associated with childhood scarcity) toward health-conscious dietary adaptations to manage age-related chronic conditions (e.g., reducing salt and fats). While retirement provides older adults with abundant time, their primary barriers are biological, driven by physical fatigue and the lack of motivation associated with cooking for oneself.

## 4 Discussion

The present findings, based on participants’ self-reported perceptions and experiences, suggest that young adults and individuals aged over 59 years may follow apparently distinct culinary trajectories, yet appear to rely on remarkably similar adaptive strategies. Rather than abandoning traditional cuisine, younger adults appear to modernize its preparation through the integration of healthy convenience products, such as canned legumes and minimally processed ingredients, together with time-saving cooking technologies, including air fryers and microwave ovens. These adaptations enable them to preserve family culinary traditions while substantially reducing meal preparation time. This interpretation is consistent with recent evidence on the domestic use of time-saving kitchen appliances, indicating that younger generations are not abandoning traditional cooking practices but are instead transforming the way they are implemented while preserving their sensory and cultural value (28). This modernization is supported by quantitative data in Spain showing a significant reliance on modern household appliances for healthy cooking methods (1).

The growing awareness of UPFs among young adults, frequently driven by a proactive orientation toward self-care and both physical and emotional well-being, is also supported by recent evidence documenting an increasing tendency to avoid these products, particularly among individuals with higher educational attainment (29, 30). Although both age groups expressed concern about dietary quality, they differed substantially in the meanings attributed to healthy eating and to limiting UPF consumption. Among younger participants, reducing UPF intake represented an intentional expression of autonomy, personal responsibility, and self-care, consistent with evidence linking high UPF consumption not only to adverse health outcomes but also to detrimental social and environmental consequences (31).

In contrast, among older participants, dietary modifications primarily reflected a clinically oriented and adaptive response to chronic disease. Traditional recipes were reformulated by reducing salt, fat, and energy density while preserving their cultural identity, suggesting that dietary change was motivated mainly by the need to manage established chronic conditions and prevent disease progression or related complications rather than by preventive intentions. Collectively, these findings indicate that the role attributed to food evolves across the life course, shifting from a predominantly preventive and value-based perspective among younger adults to a more therapeutic function in older age, in agreement with findings from real-world clinical practice studies (32). This pattern is consistent with the literature describing dietary trajectories in later life as a process of progressive “medicalization,” whereby food choices become increasingly oriented toward the management of comorbidities rather than reflecting spontaneous identity-based preferences (33). Likewise, studies investigating facilitators and barriers to the implementation of nutritional interventions among frail older adults consistently show that intrinsic motivation and social support are stronger determinants of dietary adherence than the greater availability of time typically associated with retirement (34).

Another important finding concerns the social dimension of eating. Older participants described preparing and consuming meals in the absence of companionship or social support as a monotonous and demotivating experience, whereas the presence of family members, guests, or other companions acted as a powerful facilitator of healthy eating behaviors. This observation is consistent with evidence showing that social isolation among older adults is associated with inadequate intakes of essential macronutrients, particularly protein, as well as several key micronutrients, including magnesium, potassium, folate, vitamin B6, and vitamin C (35). Recent studies have further demonstrated that eating alone is associated with less structured daily eating routines and a greater reliance on ready-to-eat or nutritionally poorer foods, resulting in lower intakes of essential micronutrients. These findings have led some authors to describe a form of “nutritional silence” affecting frail older adults living alone, highlighting their heightened vulnerability to nutritional risk (36, 37). Notably, this dietary impoverishment associated with solitude is not exclusive to older populations; quantitative evidence in Spain confirms that younger adults living alone also present a significantly lower healthy eating index and increased consumption of fast food and ultra-processed dishes compared to those living with company (38).

Within this context, commensality emerged not simply as an accompanying feature of meals but as an important protective determinant of healthy eating. Although this role was observed across all age groups, its meaning differed according to age. Among younger participants, commensality primarily reflected the social experience of sharing meals, whereas among older adults it reinforced the relational and symbolic significance of traditional dishes, particularly those based on legumes and vegetables. This finding is particularly noteworthy when interpreted alongside the international literature, where, outside Spain and the Mediterranean region more broadly, these foods are often viewed from a predominantly functional or “utilitarian” perspective (39), largely devoid of the emotional, nostalgic, and identity-related meanings that characterize Mediterranean food cultures. Within this cultural context, traditional dishes continue to function as authentic cultural markers and as vehicles for the intergenerational transmission of culinary heritage (40).

The persistence of these dishes as “culinary anchors,” capable of transcending generations despite the pressures of food globalization, therefore represents a distinctive feature of the population examined in the present study. This interpretation is further supported by the literature on “food nostalgia” (41), which describes food as a powerful trigger of childhood memories and emotionally significant family relationships, particularly those involving mothers and grandmothers. Beyond its nutritional role, food therefore contributes to emotional well-being, reinforces social connectedness, and promotes continuity of cultural identity, all of which are distinctive characteristics of Mediterranean societies.

Taken together, these findings indicate that younger and older adults experience complementary rather than overlapping barriers to healthy cooking. Younger adults display strong health motivation and social awareness but are constrained by limited time availability and insufficient practical cooking skills (3). Conversely, older adults benefit from greater culinary experience and more available time but are challenged by declining physical capacity and, in some cases, by reduced social motivation associated with living alone. Notably, this shared reliance on kitchen technology serves a dual purpose across the life course: overcoming time scarcity and skill limitations in youth, while mitigating physical fatigue and domestic labor in older age. This mirrored pattern is consistent with previous literature showing that limited time and inadequate cooking skills represent the principal barriers to home meal preparation among young adults regardless of geographical setting (3, 42, 43).

### 4.1 Home cooking and future public health perspectives

The intergenerational elements that emerged from this study point to the potential relevance of traditional home-cooked food beyond individual dietary behavior, and may inform hypotheses about its role within sustainable food systems. Given the qualitative and exploratory nature of the design, these considerations are offered as avenues for future research rather than as established determinants of chronic disease risk. International data confirm that home meal preparation represents an accessible and low-cost strategy to reduce UPF intake and improve diet quality (44). Our results suggest that young adults are redesigning rather than abandoning traditional home-cooked food, also thanks to new ways of preparing and cooking food, while older adults sustain it thanks to the experience accumulated despite the limits imposed by physical and emotional decline. Public health strategies should favor specific interventions in each stage of life, favoring generational integration (45).

A further aspect of relevance for food policy is the promotion of traditional legume-based dishes that are characteristic of the Mediterranean dietary pattern, as they combine nutritional benefits, environmental sustainability, and cultural value. In addition to representing a culinary heritage that should be preserved and transmitted across generations, these traditional preparations provide a high-quality source of plant protein, characterized by a lower environmental footprint and greater economic affordability than many alternative protein sources (46). The convergence of these attributes positions legumes as a strategic component of public policies aimed at promoting healthy and sustainable food systems by simultaneously advancing public health, environmental protection, and equity in access to healthy diets (47–49).

Another important public health consideration concerns the role of social isolation and loneliness as determinants of nutritional risk, particularly among older adults and, more broadly, among populations experiencing socioeconomic vulnerability and social frailty. In these settings, community-based interventions such as shared meal programs, intergenerational cooking initiatives, and local networks supporting the preparation and sharing of traditional dishes may represent cost-effective strategies to simultaneously improve diet quality, social participation, and psychosocial well-being, while complementing conventional approaches based on individual nutritional counseling (50). Integrating these interventions into care pathways for individuals living with frailty, multimorbidity, and complex health and social care needs could help address both nutritional inadequacy and social isolation, two conditions that frequently coexist and reinforce one another. Although the greatest benefits are likely to be observed among older adults, positive effects may also extend to younger generations by strengthening intergenerational cohesion and social connectedness (51).

Taken together, these strategies outline a health promotion model that recognizes traditional home cooking as both a cultural asset and a public health resource. From this perspective, the intergenerational transmission of culinary knowledge, cooking skills, and food practices, supported by digital technologies for nutrition education and social engagement, could represent a strategic lever to enhance food system resilience, promote healthy and sustainable dietary patterns, and preserve communities’ food cultural heritage (52).

### 4.2 Limitations

This study has several methodological limitations that should be considered when interpreting the findings. The cross-sectional qualitative design, based on open-ended responses collected through an online questionnaire, did not allow the level of interpretative depth typically achievable through semi-structured interviews or focus groups, thereby limiting the iterative exploration of emerging themes and a more in-depth understanding of the meaning participants attributed to their experiences. The data collection approach precluded the application of the conventional criterion of theoretical saturation, leading instead to the adoption of a retrospective assessment of thematic sufficiency. Although this approach has recently been proposed in the methodological literature for qualitative studies using open-ended survey responses, it may be less sensitive in capturing minority or highly heterogeneous perspectives. Furthermore, the older cohort (*n* = 52) was twice as large as the younger cohort (*n* = 26). Although this reflects the natural age distribution of the source sample and all valid responses were retained to maximize descriptive richness, the asymmetry may have exerted an uneven weighting on the comparative themes and should be considered when interpreting the intergenerational contrasts.

In addition, recruitment through digital channels and social media may have introduced selection bias, with the likely overrepresentation of individuals with higher digital literacy, greater interest in food-related topics, and a higher propensity to participate in online research. Consequently, the findings may not be fully transferable to the broader Spanish population, particularly to socially or geographically disadvantaged groups with more limited access to or engagement with digital platforms. Another limitation relates to the self-reported nature of the data, which precluded verification of the correspondence between reported culinary behaviors and actual food preparation practices, thereby introducing the potential influence of social desirability and recall bias.

Finally, the lack of clinical and social care characterization, particularly within the older adult cohort, limits the extent to which the findings can be transferred to specific subgroups of the older population, such as individuals living with frailty, multimorbidity, functional limitations, or varying levels of social support, whose needs, motivations, and food-related behaviors may differ substantially.

## 5 Conclusion

Within the limits of a qualitative, exploratory design, this study identified, based on participants’ self-reported accounts, generational differences in cooking practices and in the value attributed to traditional cuisine, suggesting lower perceived cooking skills and greater time-related barriers among younger adults. These findings indicate that traditional cuisine represents not only a cultural heritage but also a potential resource for promoting health, sustainability, and social cohesion. Further longitudinal and intervention studies are warranted to determine whether strengthening cooking skills and promoting traditional food practices can translate into measurable improvements in nutritional, clinical, and social outcomes. At the policy level, these findings may support the consideration of traditional cuisine as a potential component of public health strategies that integrate nutrition education, technological innovation, and the preservation of cultural heritage, although confirmatory research is needed before drawing firm policy conclusions.

## Data Availability

The raw data supporting the conclusions of this article will be made available by the authors, without undue reservation.
