# Supplementary material for: Culinary memories, perceived barriers, and motivations for home cooking among younger and older adults in Spain: a qualitative cross-sectional study
Source: Front Nutr. 2026 Sep 24;13:1938313. doi: 10.3389/fnut.2026.1938313 (PMC13647459; doi:10.3389/fnut.2026.1938313)
Supplement: Supplementary file 1 [file Table_1.DOCX]

**Supplementary material S1**

**Consolidated criteria for reporting qualitative studies (COREQ): 32-item checklist**

*Developed from:*

Tong A, Sainsbury P, Craig J. Consolidated criteria for reporting qualitative research (COREQ): a 32-item checklist for interviews and focus groups. International Journal for Quality in Health Care. 2007. Volume 19, Number 6: pp. 349–357

**MANUSCRIPT TITLE:** ______________________________________________________________

| **No. Item** | **Guide questions/description** | **Reported in Section** |
| --- | --- | --- |
| **Domain 1: Research team and reflexivity** | | |
| ***Personal Characteristics*** | | |
| **1. Interviewer/facilitator** | Which author/s conducted the interview or focus group? | N/A – online survey (2.3, 2.3.1) |
| **2. Credentials** | What were the researcher’s credentials? (E.g. PhD, MD) | 2.1 |
| **3. Occupation** | What was their occupation at the time of the study? | 2.1 |
| **4. Gender** | Was the researcher male or female? | 2.1 |
| **5. Experience and training** | What experience or training did the researcher have? | 2.1 / 2.5 |
| ***Relationship with participants*** | | |
| **6. Relationship established** | Was a relationship established prior to study commencement? | N/A – anonymous online survey (2.3, 2.3.1) |
| **7. Participant knowledge of the interviewer** | What did the participants know about the researcher? (e.g. personal goals, reasons for doing the research) | 2.2 / 2.3.1 |
| **8. Interviewer characteristics** | What characteristics were reported about the interviewer/facilitator? (e.g. Bias, assumptions, reasons and interests) | 2.6 |
| **Domain 2: Study design** | | |
| ***Theoretical framework*** | | |
| **9. Methodological orientation and Theory** | What methodological orientation was stated to underpin the study? | 2.1 / 2.5 |
| ***Participant selection*** | | |
| **10. Sampling** | How were participants selected? (e.g. purposive, convenience, consecutive, snowball) | 2.4 / 2.4.1 |
| **11. Method of approach** | How were participants approached? (e.g. face-to-face, telephone, mail, email) | 2.3.1 |
| **12. Sample size** | How many participants were in the study? | 2.4 / 3.1 |
| **13. Non-participation** | How many people refused to participate or dropped out? Reasons? | 2.3.1 / 2.4 |
| ***Setting*** | | |
| **14. Setting of data collection** | Where was the data collected? (e.g. home, clinic, workplace) | 2.3 / 2.3.1 |
| **15. Presence of non-participants** | Was anyone else present besides the participants and researchers? | N/A – self-administered online survey (2.3) |
| **16. Description of sample** | What are the important characteristics of the sample? (e.g. demographic data, date) | 2.4 (Table 1) / 3.2 (Table 2) |
| ***Data collection*** | | |
| **17. Interview guide** | Were questions, prompts, guides provided by the authors? Was it pilot tested? | 2.3 (Supplementary File 2) |
| **18. Repeat interviews** | Were repeat interviews carried out? If yes, how many? | N/A – single-submission survey (2.3.1) |
| **19. Audio/visual recording** | Did the research use audio or visual recording to collect the data? | N/A – text-based survey (2.3) |
| **20. Field notes** | Were field notes made during and/or after the interview or focus group? | N/A – online survey (2.3) |
| **21. Duration** | What was the duration of the interviews or focus group? | 2.3 (data collection period Nov 2025–Jan 2026) |
| **22. Data saturation** | Was data saturation discussed? | 2.7 / 2.8 |
| **23. Transcripts returned** | Were transcripts returned to participants for comment and/or correction? | N/A – anonymous survey (2.6) |
| **Domain 3: Analysis and findings** | | |
| ***Data analysis*** | | |
| **24. Number of data coders** | How many data coders coded the data? | 2.5 |
| **25. Description of the coding tree** | Did authors provide a description of the coding tree? | 2.5 (Table 2) / 3.3 / 3.5 |
| **26. Derivation of themes** | Were themes identified in advance or derived from the data? | 2.5 |
| **27. Software** | What software, if applicable, was used to manage the data? | 2.5 |
| **28. Participant checking** | Did participants provide feedback on the findings? | 2.6 |
| ***Reporting*** | | |
| **29. Quotations presented** | Were participant quotations presented to illustrate the themes/findings? Was each quotation identified? | 3.4 / 3.6 |
| **30. Data and findings consistent** | Was there consistency between the data presented and the findings? | 3.3 / 3.5 / 4 |
| **31. Clarity of major themes** | Were major themes clearly presented in the findings? | 3.3 / 3.5 / 3.7 |
| **32. Clarity of minor themes** | Is there a description of diverse cases or discussion of minor themes? | 3.4 / 3.6 / 4.1 |

**Supplementary Material S2**

*Survey instrument: socio-demographic items and open-ended questions*

This supplementary file reproduces the survey instrument used in the present study. It comprises the socio-demographic section and the five open-ended qualitative questions on which the qualitative content analysis was based. Questions are presented in the original Spanish (the language of administration) together with an English translation. The instrument was completed online; participation was voluntary and anonymous, and access to the questionnaire required prior informed consent.

## 1. Informed consent (summary)

Before accessing the questionnaire, all respondents were presented with a mandatory informed-consent screen stating the purpose and nature of the research, the identity of the research team, the right to withdraw at any time without justification or penalty, and confirmation that the data would be used exclusively for academic and research purposes. Consent was granted digitally by selecting “Yes, I wish to participate” before any survey item was displayed.

## 2. Socio-demographic items

| **Variable (ES)** | **Variable (EN)** |
| --- | --- |
| Sexo | Sex |
| Edad | Age |
| Lugar de residencia | Place of residence |
| Nivel de estudios completado | Educational level completed |
| Estudios relacionados con la salud (sí/no) | Health-related studies (yes/no) |
| Nivel de ingresos netos actuales del hogar | Current household net income level |
| Nivel de ingresos del hogar en la infancia | Household income level during childhood |

## 3. Open-ended qualitative questions

*The following five open-ended questions correspond to those reported in Table 1 of the main manuscript (one transition question, three key questions, and one ending question).*

**Q1. *Transition question / Pregunta de transición***

**[ES]** Para muchas personas hay algunas comidas que les recuerdan más a su infancia o a su familia. ¿Hay alguna receta que siga preparando porque le recuerda a su infancia? ¿Cuál es y por qué? ¿Recuerda algún sabor o receta que ya no haya vuelto a experimentar? ¿Qué significaba para usted?

**[EN]** *For many people, certain meals strongly remind them of their childhood or family. Is there a recipe that you still prepare because it reminds you of your upbringing? What is it and why? Do you remember any specific flavour or recipe that you haven't experienced again? What did it mean to you?*

**Q2. *Key question / Pregunta clave***

**[ES]** ¿Qué recuerdos o experiencias personales tiene, en positivo y en negativo, de la cocina en su infancia? ¿Cómo se cocinaba en su casa? ¿En qué se diferencia de la que hace ahora en su casa?

**[EN]** *What personal memories or experiences, both positive and negative, do you have regarding cooking during your childhood? How was cooking done in your home? How does it differ from the way you cook in your home now?*

**Q3. *Key question / Pregunta clave***

**[ES]** ¿Nos puede decir si hay platos a los que no renunciaría nunca y por qué?

**[EN]** *Could you tell us if there are any dishes you would never give up, and why?*

**Q4. *Key question / Pregunta clave***

**[ES]** ¿Ha hecho cambios en su forma de cocinar en los últimos años que crea que han mejorado o empeorado su salud o los de la unidad familiar? ¿Qué tipo de cocina considera “saludable”? ¿Por qué?

**[EN]** *Have you made changes in the way you cook in recent years that you believe have improved or worsened your health or that of the family unit? What type of cooking do you consider “healthy”? Why?*

**Q5. *Ending question / Pregunta de cierre***

**[ES]** ¿Qué opina sobre los platos preparados, la comida para llevar, etc.? ¿Qué pros y qué contras le ve respecto de cocinar en el hogar?

**[EN]** *What is your opinion about ready-made meals, takeaway food, etc.? What advantages and disadvantages do you see compared with home cooking?*

**Note.** *The survey formed part of a broader research instrument; only the items analysed in the present study are reproduced here.*
